# How life events may confer vulnerability to addiction: the role of epigenetics

**DOI:** 10.3389/fnmol.2024.1462769

**Published:** 2024-09-18

**Authors:** Shirelle X. Liu, Andrew C. Harris, Jonathan C. Gewirtz

**Affiliations:** ^1^Department of Psychology, University of Minnesota, Minneapolis, MN, United States; ^2^Department of Medicine, University of Minnesota, Minneapolis, MN, United States; ^3^Hennepin Healthcare Research Institute, Minneapolis, MN, United States; ^4^Department of Psychology, Arizona State University, Tempe, AZ, United States

**Keywords:** substance use disorder, epigenetics, environment, vulnerability, animal models

## Abstract

Substance use disorder (SUD) represents a large and growing global health problem. Despite the strong addictive potency of drugs of abuse, only a minority of those exposed develop SUDs. While certain life experiences (e.g., childhood trauma) may increase subsequent vulnerability to SUDs, mechanisms underlying these effects are not yet well understood. Given the chronic and relapsing nature of SUDs, and the length of time that can elapse between prior life events and subsequent drug exposure, changes in SUD vulnerability almost certainly involve long-term epigenetic dysregulation. To validate this idea, functional effects of specific epigenetic modifications in brain regions mediating reinforcement learning (e.g., nucleus accumbens, prefrontal cortex) have been investigated in a variety of animal models of SUDs. In addition, the effects of epigenetic modifications produced by prior life experiences on subsequent SUD vulnerability have been studied, but mostly in a correlational manner. Here, we review how epigenetic mechanisms impact SUD-related behavior in animal models and summarize our understanding of the relationships among life experiences, epigenetic regulation, and future vulnerability to SUDs. Despite variations in study design, epigenetic modifications that most consistently affect SUD-related behavior are those that produce predominantly unidirectional effects on gene regulation, such as DNA methylation and histone phosphorylation. Evidence explicitly linking environmentally induced epigenetic modifications to subsequent SUD-related behavior is surprisingly sparse. We conclude by offering several directions for future research to begin to address this critical research gap.

## 1 Introduction

Substance use disorders (SUDs) impose severe burdens on individuals and society (Sharma et al., 2019; Strang et al., 2020; Ignaszewski, 2021; Peterson et al., 2021). Despite the addictive potency of drugs of abuse, a large majority of the population that experiments with drugs does not develop SUDs (Vowles et al., 2015; Reynolds et al., 2021). The trajectory that leads some into a SUD and others to avoid it is assumed to be a product of heritable variations in DNA sequence (e.g., single nucleotide and structural variants) and environmental factors, including an individual’s current circumstances and the cumulative effects of their past life experiences. With regard to the latter, epidemiological research has documented the persistent and cumulative effects of multiple adverse circumstances that increase susceptibility to SUDs and other psychopathologies. Most notable among them is the enduring impact of trauma, especially when experienced during critical developmental periods (Alati et al., 2006; Radliff et al., 2012; Zarse et al., 2019).

**FIGURE 1:**
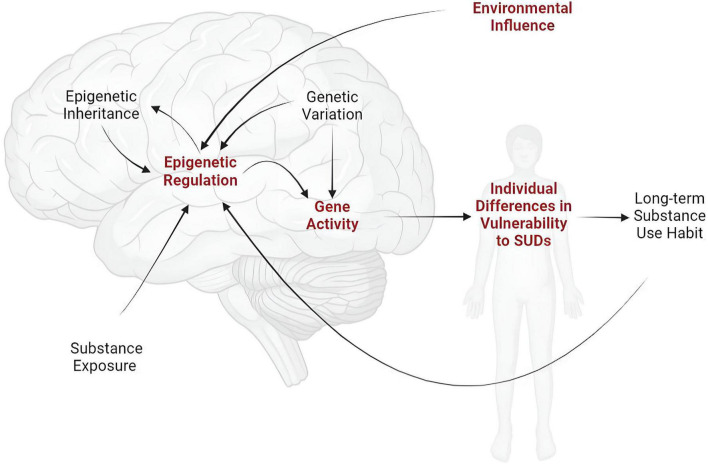
Individual vulnerability to SUDs is shaped by inherited genetic variation and by current and past environmental experiences, including substance exposure. Given the enduring nature of SUDs, these effects are almost certainly produced and sustained mechanistically through molecular pathways that regulate gene expression (i.e., epigenetics). While research has focused intensively and extensively on the effects of genetic variation and substance exposure and use on gene expression, relatively little is known about the epigenetic mechanisms through which long-term environmental factors affect susceptibility to SUDs (indicated in red).

The long-term nature of such effects implicates epigenetic adaptation - or molecular regulation of gene transcription - within the brain as an underlying mechanism (Mews et al., 2018; Hamilton and Nestler, 2019; Werner et al., 2021). Some studies have documented epigenetic changes resulting from prior life experiences, such as early-life drug exposure or stress, while others have implicated epigenetic modifications in the addictive effects of drugs of abuse. Surprisingly few studies, however, have demonstrated a causal relationship between the presumed epigenetic “scars” left by major life events and future vulnerability to SUDs (Figure 1).

While human studies have been critical in implicating epigenetic mechanisms in SUD vulnerability, variations in factors such as stress exposure, parenting styles, the severity of drug use, or developmental period (e.g., prenatal, adolescent), often make it difficult to characterize the finer details of relationships between specific forms of experience and SUD-related outcomes. Furthermore, epigenetic variance is tissue-specific, which can only be assayed in human brain tissue post-mortem. The harvesting of animal brain tissue, clearly, is less subject to this constraint. Furthermore, animal studies are designed to be fully experimental, randomized, and controlled, allowing greater precision and specificity in evaluating the effects of environmental factors or of different levels of drug exposure on epigenetic changes and SUD vulnerability.

A variety of preclinical behavioral models of SUDs have been developed (Table 1). Among these, drug self-administration (SA) is often considered the “gold standard” for measuring SUD-like behavior because it involves volitional drug taking as occurs in humans and can be used to model various phases of the SUD trajectory (e.g., acquisition, relapse, etc., see Table 2). However, data from other models (e.g., conditioned place preference, CPP) can also provide important insights into the mechanisms underlying SUD. In this review, we will outline the forms of epigenetic modification that regulate SUD-related behavior in animals, summarize our understanding of their roles in mediating environmentally driven vulnerability to SUDs, and highlight several promising future research directions.

**TABLE 1:** Behavioral measures.

Model	Definition
Drug self-administration	An operant conditioning procedure in which animals perform a response (e.g., lever press) in order to obtain a drug delivered intravenously or orally. Higher levels of responding for a drug compared to its vehicle measure the drug’s primary reinforcing effects. Procedural variations such as alterations in duration of access, schedule of reinforcement, or presentation versus omission of drug-associated cues can provide measures of multiple facets of addictive behavior.
Conditioned place preference	A Pavlovian conditioning procedure in which animals are confined to one chamber following noncontingent drug exposure, and another distinct chamber following vehicle exposure. During testing, animals are allowed to freely explore both chambers. Increased time spent in the drug-paired chamber compared to the vehicle-paired chamber measures the drug’s conditioned rewarding effects.
Locomotor sensitization	A progressive increase in a drug’s locomotor stimulant effects across repeated drug exposures

**TABLE 2:** Measures of drug SA.

Stage of SUD	SA model	Operational measure
Initiation of drug use	Acquisition	Average number of infusions earned during first days of drug SA
Reinforcing efficacy	Progressive ratio schedule of reinforcement	Breakpoint, or the highest fixed ratio at which the animal maintains responding for drug
Loss of control over drug use	Escalation	Increase in rate of infusions earned after duration of daily access to drug is extended
Drug use despite negative consequences	Resistance to punishment	Reduction in drug SA when infusions are accompanied by aversive consequence (e.g., foot shock)
Cessation	Extinction	Reduction in drug-seeking when a self-administered drug is replaced with its vehicle
Relapse to drug use following exposure to drug-associated environmental cues, stress, or the drug itself	Cue-/stress-/drug-induced reinstatement	Increase in drug-seeking (active lever pressing) following extinction of SA and exposure to drug-associated cue stimuli, stress (e.g., foot shock), or non-contingent injection of previously self-administered drug

Reprinted with modifications from “Behavioral predictors of individual differences in opioid addiction vulnerability as measured using i.v. self-administration in rats. Swain et al. (2021)” with permission from Elsevier).

## 2 Epigenetic modifications mediating addictive effects of substances

Epigenetic regulation affects both transcriptional and post-transcriptional processes, with the latter including alternative splicing and RNA silencing (Wong et al., 2011). Such regulation occurs through a complex set of molecular processes, including DNA and histone modification, chromatin remodeling, and the actions of non-coding RNAs (ncRNAs). While preclinical studies have enumerated ways in which chromosomal modifications are up- or down-regulated by exposure to addictive substances, few have examined the causal effects of specific forms of epigenetic modification on SUD-related behavior. As described below, and summarized in Supplementary Table 1, such studies have produced complex and often inconsistent results.

### 2.1 DNA modification

#### 2.1.1 Molecular mechanisms

Covalent modifications of the nucleotide bases of DNA regulate gene transcription without altering the genetic code itself. The most commonly studied form is 5-methylcytosine (5mC), which occurs through DNA methyltransferase (DNMT) enzymes. 70% of 5mC moieties in the adult brain are found on cytosine-guanine (CpG) sites (Lister et al., 2013). CpG-rich regions of around 1,000 base-pairs (bp), CpG islands (CGIs), contain 70% of gene promoters and are typically hypomethylated (6–8% methylation) (Illingworth et al., 2008). Methylation at CGIs commonly reduces binding of transcription factors (TFs), thereby suppressing gene expression (Deaton and Bird, 2011). Conversely, 5mCs located at the gene body are associated with gene activation and alternative splicing (Jones, 2012).

A second DNA modification is 5-hydroxymethylcytosine (5hmC), formed through the oxidation of 5mC by ten-eleven translocation methylcytosine dioxygenase (TET) enzymes (Goll and Bestor, 2005; Ito et al., 2011). 5hmC is highly enriched in the CNS (∼17% of the methylated genome) relative to the periphery (Lister et al., 2013). While the functions of hydroxymethylation are complex, promotion of gene expression is the most prominent (Ehrlich and Ehrlich, 2014). 5hmC is sequentially converted to 5-formyl and 5-carboxyl cytosine, before its eventual dissociation from cytosine residues (Shi et al., 2017).

#### 2.1.2 Behavioral effects

The effects of systemic or brain region-specific demethylation through DNMT inhibitors (DMNTis) on behaviors associated with addictive drugs have been fairly consistent. Most studies report that reduced DNA methylation decreased animals’ locomotor sensitivity, drug SA (e.g., cue- and drug-induced reinstatement; see Table 2), or conditioned place preference (CPP; see Supplementary Table 1) for cocaine, opioids or alcohol (Anier et al., 2010; Han et al., 2010; Warnault et al., 2013; Barbier et al., 2015; Massart et al., 2015; Hong et al., 2021) (but see Laplant et al., 2010). In contrast, increasing methylation through methyl donors has yielded mixed results, depending on the donor administered and the substance studied. While systemic injection of the methyl donor methionine (MET) decreased rodents’ locomotor sensitivity, SA (drug-induced reinstatement) or CPP for cocaine (Laplant et al., 2010; Tian et al., 2012; Wright et al., 2015), S-adenosylmethionine (SAM) has opposite effects (Anier et al., 2013; Massart et al., 2015). Similarly, systemic administration of MET increased opioid SA (drug-induced reinstatement) in one study but had no significant effect on opioid CPP in another (Tian et al., 2012; Hong et al., 2021). In addition, altering the balance between methylation and hydroxymethylation through knockdown of the TET1 enzyme in the nucleus accumbens (NAc), increased CPP for cocaine in mice, consistent with the changes induced by repeated cocaine administration (Feng et al., 2015).

### 2.2 Histone modification

The basic structural and functional unit of chromatin is the nucleosome, composed of ∼147-bp DNA wrapped around an octamer containing 2 copies each of the histones H2A, H2B, H3, and H4 (Andrews and Luger, 2011). Post-translational histone monomers can be covalently modified to incorporate various functional groups at their N-terminals. At least 18 different forms of histone modification have been identified, most commonly at lysine (K) residues on histones H3 and H4 (Zhao and Garcia, 2015). Among these, acetylation, methylation, and phosphorylation have been most frequently studied in behavioral models of SUD.

#### 2.2.1 Histone acetylation

##### 2.2.1.1 Molecular mechanisms

Acetylation of lysine residues of the N-terminal histone tail is closely associated with transcriptional activation. While thought to encourage recruitment of chromatin remodeling proteins, the causal roles of acetylation in gene transcription are still not well established. For example it has still to be determined whether the strongest indicator of transcriptional activation, H3K27ac, is a transcriptional effector itself or simply provides a close readout of transcriptional activation (Shvedunova and Akhtar, 2022). Histone acetyltransferases (HATs) and histone deacetylases (HDACs) mediate histone acetylation and deacetylation, respectively (Shahbazian and Grunstein, 2007).

##### 2.2.1.2 Behavioral effects

Stimulants tend to increase acetylation of histones (Renthal and Nestler, 2009; McCowan et al., 2015). Upregulation of histone acetylation by other means has been shown to enhance stimulant-associated behaviors in most (Levine et al., 2005; Kalda et al., 2007; Renthal et al., 2007; Shen et al., 2008; Sun et al., 2008; Wang et al., 2010; Malvaez et al., 2011; Rogge et al., 2013; Campbell et al., 2021), but not all studies (Romieu et al., 2008; Ferguson et al., 2013; Kennedy et al., 2013; Taniguchi et al., 2017; Campbell et al., 2021). Notably, Campbell et al. (2021) demonstrated that the same manipulation could produce opposite effects depending on the specific behavioral paradigm: reducing HDAC3 activity in NAc dopamine receptor D1 (DRD1)-containing medium spiny neurons decreased cocaine seeking during extinction of SA (see Table 2), but enhancing cocaine-CPP. In contrast, increased histone acetylation inhibits alcohol- and nicotine-associated behaviors, including voluntary alcohol intake (Warnault et al., 2013; Sakharkar et al., 2014; Bohnsack et al., 2022), alcohol withdrawal-induced anxiety-like behavior (Sakharkar et al., 2014; Bohnsack et al., 2022), and nicotine CPP (Pastor et al., 2011). The effects of altering histone acetylation on opioid addiction have been the least conclusive, with behavioral effects in both directions (Ferguson et al., 2013; Chen et al., 2016; Saberian et al., 2021; Anderson et al., 2023).

#### 2.2.2 Histone phosphorylation

##### 2.2.2.1 Molecular mechanisms

Like histone acetylation, phosphorylation reduces the positive charge on histone molecules thereby promoting gene expression. Mediated by protein kinases, histone phosphorylation most frequently occurs at serine (S) residues on the histone N-terminal tails, but also at threonine (T) and tyrosine (Y) residues (Rossetto et al., 2012). Histone phosphorylation at certain sites is closely associated with nearby acetylation, a process referred to as phosphoacetylation (Clements et al., 2003; Rossetto et al., 2012). For example, phosphorylation at H3S10, H3T11 and H3S28 facilitates acetylation at H3K14 by forming a HAT/CoA/histone complex, leading to increased HAT activity (Lo et al., 2000).

##### 2.2.2.2 Behavioral effects

Few studies have specifically investigated the effects of histone phosphorylation or phosphoacetylation on SUD-associated behavior, although an increase in histone phosphorylation or phosphoacetylation most commonly enhances SUD-related behavior (Kumar et al., 2005; Stipanovich et al., 2008; Besnard et al., 2011; Sheng et al., 2011). Conversely, a reduction of histone H3 phosphorylation and phosphoacetylation decreased cocaine-induced locomotor sensitization but enhanced cocaine-CPP (Brami-Cherrier et al., 2005). In addition, some research has demonstrated that pharmacologically inhibiting extracellular signal-regulated kinases 1 and 2 (ERK1/2), which phosphorylate a wide range of proteins including histones and their epigenetic modifiers, generally reduce behavioral responses to psychostimulants and marijuana (Pascoli et al., 2014; Sun et al., 2016).

#### 2.2.3 Histone methylation

##### 2.2.3.1 Molecular mechanisms

Histone methylation most frequently occurs at lysine and arginine (R) sites. Mediated by histone N-methyltransferases (HMTs), lysine residues can be mono-, di-, or trimethylated, and arginine residues can be mono- or di-methylated (Miller and Grant, 2013). Methyl groups can be removed by lysine-specific histone demethylases (KDMs) and arginine demethylases (Bannister and Kouzarides, 2003; Greer and Shi, 2012). Histone methylation is more stable than either acetylation or phosphorylation, suggesting longer-lasting effects on gene regulation (Zee et al., 2010; Mews et al., 2014). In contrast to the permissive effects of histone acetylation and phosphorylation, histone methylation can either permit or repress gene transcription, depending on the target residue, its degree of methylation and, in some cases, the presence of other marks in its vicinity (Kouzarides, 2002). For example, H3K4me3 (permissive) and H3K27me3 (repressive) marks interact bivalently, presenting a potential target for studying dynamic gene regulation in response to changes in environmental conditions (Blanco et al., 2020).

##### 2.2.3.2 Behavioral effects

Given their complex nature, it is not surprising that the effects of histone methylation on SUD-related behavior are not uniform. For example, approximately half of the studies on psychostimulants have found that an increase in a permissive histone methylation mark (e.g., H3K4me3, H4R3me2a) or decrease in a repressive mark (e.g., H3K9me2) heightens responses to stimulants (Maze et al., 2010; Kennedy et al., 2013; Aguilar-Valles et al., 2014; Heller et al., 2014; Li et al., 2015; Zhang et al., 2018), while the remainder have reported the reverse (e.g., permissive: H3K4me3; repressive: H3K9me2, H3K36me3, H3R2me2a) (Aguilar-Valles et al., 2014; Damez-Werno et al., 2016; Anderson et al., 2018b,2018a; Xu et al., 2021). One study suggested that decreased enrichment of a repressive methylation mark (H3K9me1) led to increased alcohol consumption (Barbier et al., 2016). Moreover, HDACi increased both histone acetylation and repressive methylation, leading to decreased locomotor sensitization to cocaine (Kennedy et al., 2013). This example shows the complicated interaction between chromatin marks with different functions.

### 2.3 Chromatin remodeling

#### 2.3.1 Molecular mechanisms

Repositioning of nucleosomes causes surrounding chromatin to assume more open or closed configurations that promote or depress gene expression, respectively. Changes in DNA and histone modification can induce alterations in chromatin structure by recruiting ATPase-containing chromatin remodeling enzymes (SWI/SNF, CHD, ISWI and INO80 families, Längst and Manelyte, 2015).

#### 2.3.2 Behavioral effects

Promotion of open-chromatin heightens locomotor sensitization and drug preference for cocaine (Wang et al., 2016; Salery et al., 2017; Werner et al., 2019). These changes in chromatin conformation have been achieved by altering the abundance and functionality of the chromatin remodelers SWI/SNF and INO80, and of activity-regulated cytoskeleton-associated protein, which interacts with the SWI/SNF complex (Shan et al., 2020; Hargreaves, 2021). Results of this approach have not yet been reported for other addictive substances.

### 2.4 ncRNAs

ncRNAs comprise microRNAs (miRNAs), small nucleolar RNAs (snoRNAs), circular noncoding RNAs (circRNAs) and long noncoding RNAs (lncRNAs). Unlike mRNAs that serve as templates for translation, these molecules regulate gene transcription (Chekulaeva and Rajewsky, 2019).

#### 2.4.1 miRNA: molecular mechanisms and behavioral effects

Among ncRNAs, the role of miRNAs in the various effects of addictive drugs are the best understood. miRNAs are ∼22-nucleotide (nt)-long molecules that can form an RNA-induced silencing complex (RISC) to bind to complementary target mRNAs (Shang et al., 2023). This process induces cleavage of the mRNA transcript and inhibition of protein translation, affecting SUD-like behavior, possibly through alterations in synaptic signaling (Bartel, 2004; Chandrasekar and Dreyer, 2009; Jonkman and Kenny, 2012; Kenny, 2022). This mechanism has been explored extensively in animal models of cocaine use. Conditional knockout of RISC protein AGO2 in Drd2 neurons abolished the acquisition of cocaine SA (acquisition) and CPP in mice (Schaefer et al., 2010). In addition, overexpression of each of the miRNAs miR-124, -212, -495, and let-7d reduced behavioral responses to cocaine (Hollander et al., 2010; Chandrasekar and Dreyer, 2011; Bastle et al., 2017), while overexpression of miR-181a enhanced cocaine-CPP (Chandrasekar and Dreyer, 2011). Interestingly, the effects of miRNA may be at least in part cell-type-specific, since overexpression of miR-1 in Drd1 neurons increased cocaine SA (cue-induced reinstatement), while overexpression in Drd2 neurons reduced cocaine SA (breakpoint) (Forget et al., 2021). In studies of alcohol use disorder, elevated levels or activity of miR-30a-5p, miR-124a, miR-137, miR-206 and miR-411 increased consumption, whereas overexpression of let-7d decreased consumption (Bahi and Dreyer, 2013, 2020; Darcq et al., 2014; Tapocik et al., 2014; Kyzar et al., 2019; Most et al., 2019).

#### 2.4.2 snoRNA: molecular mechanisms and behavioral effects

snoRNAs range from 60 to 300 nt in length and carry out diverse cellular functions (Fafard-Couture et al., 2021). While their major function is to guide chemical modifications of the target precursor RNA (pre-RNA) molecules, snoRNAs can also affect alternative splicing of pre-RNAs through complementary binding (Kiss, 2002). Likely by promoting incorporation of exon Vb into the serotonin receptor 2C (5HT2CR) transcript, overexpression of one such molecule, MBII-52, attenuated cocaine-induced CPP and locomotor sensitization (Chen et al., 2014).

#### 2.4.3 circRNA: molecular mechanisms and behavioral effects

circRNAs form a covalent loop structure through a non-canonical “back splicing” process (Rybak-Wolf et al., 2015). They are abundantly expressed, especially in the brain. circRNAs are transcribed from the same parent gene as their relevant mRNAs; however, their circular structure makes them resistant to exonuclease degradation. Additionally, circRNAs can be recognized by miRNAs that also target their respective mRNAs. As such, circRNAs may affect gene activity by protecting mRNAs from cleavage (Jeck et al., 2013). Overexpression of the circRNA circTmeff-1 in the NAc core enhanced morphine-CPP in mice while circTmeff-1 knockdown produced the opposite effect (Yu et al., 2021). Similarly, knockdown of circTmeff-1 in mouse NAc core reduced cocaine-CPP, potentially through regulation of miR-206 (Shen et al., 2022).

#### 2.4.4 lncRNA: molecular mechanisms and behavioral effects

Finally, untranslated linear RNA transcripts greater than 200 nt in length are defined as lncRNAs. Widely distributed in various tissue types, lncRNAs regulate either proximal (cis) genes or distal (trans) genes. lncRNAs can affect chromatin structure by binding with nucleosomes, neutralizing histone charges and recruiting chromatin modifiers (Statello et al., 2020). In two addiction-related studies of lncRNA function to date, overexpression of lncRNA Gas5 in the NAc decreased cocaine-CPP and SA (e.g., breakpoint, extinction) in mice (Xu et al., 2020), and knockdown of the lncRNA Lrap increased alcohol intake in rats (Saba et al., 2021).

### 2.5 Conclusion

The above studies indicate that epigenetic modifications can regulate behaviors induced by addictive drugs, but the direction of many effects has differed across studies (Table 2). Given the inherent complexity of the epigenetic regulation and the variation among study designs (e.g., behavioral paradigm, dosing regimen, manipulation method, brain region), such inconsistencies are not surprising. A further examination of the variables involved in these studies points out several that could contribute to such inconsistency. First, research on different substances produces behavioral results with different levels of consistency. For example, opioids have produced more mixed results compared to cocaine, alcohol, and nicotine. Secondly, the choice of epigenetic manipulation method can affect the behavioral outcome. For example, although reducing DNA methylation by administering DNMTi produced fairly consistent results, attempts to increase DNA methylation by injecting methyl donors (e.g., MET, SAM) failed to do so. Thirdly, the specificity of the sites where these manipulations occur (e.g., brain regions, cell types) can sometimes produce contrasting effects (Forget et al., 2021). Lastly, even when other variables are held constant, the choices of behavioral paradigm can still affect behavioral outcomes (Campbell et al., 2021). However, it is noteworthy that epigenetic modifications that have largely unidirectional effects on gene transcription, such as DNA methylation and histone phosphorylation, generally produce more consistent effects on SUD-related behaviors.

## 3 Environment and vulnerability to SUDs: role of epigenetics

Epidemiological analyses have revealed strong associations between significant early-life events, such as substance exposure and childhood trauma, on the incidence of SUDs (Enoch, 2011; McCabe et al., 2022). In the absence of a viable alternative, the most plausible mechanism is that environmental stimulation stably alters the epigenetic regulation of gene expression to affect physiological and psychological responses upon subsequent exposure to addictive substances. These changes could further lead to development and maintenance of SUDs or increase the risk of relapse after abstinence. In this section, we review studies employing animal models that have sought to establish the mechanistic relationships among environment, epigenome, and future SUD-related behavior from several perspectives.

### 3.1 Substance exposure across developmental stages

#### 3.1.1 Parental substance exposure and epigenetic inheritance

An adverse preconception environment may produce long-lasting effects on offspring (intergenerational) and future generations (transgenerational). In clinical studies, smoking and drinking in fathers are associated with adverse physical and psychological outcomes in the offspring, including asthma, sleep problems, anxiety, depression, and behavioral dysfunction (Svanes et al., 2017; Luan et al., 2022). More surprisingly, grandparental tobacco and alcohol use has been linked to neuropsychological problems in grandchildren with sex-specific effects, suggesting transgenerational inheritance (Golding et al., 2017; Kendler et al., 2018). Implicating a specific epigenetic mechanism, fathers’ preconception smoking, particularly in puberty, is associated with differential DNA methylation in the offspring of genes associated with asthma, inflammation, bipolar disorder, and binge eating (McElroy et al., 2018; Kitaba et al., 2023). This points to the possibility of environmentally induced epigenetic modifications affecting SUD vulnerability in one or more familial generations, consistent with recent evidence of the persistence of transgenerational epigenetic marks despite epigenome-wide reprogramming during gametogenesis and fertilization (Takahashi et al., 2023).

In animal studies, this has been tested primarily in sires to eliminate *in utero* effects on fetal development caused by maternal preconception substance exposure (Lassi et al., 2014). Prolonged cocaine SA in male rats during spermatogenesis reduced cocaine consumption exclusively in male offspring, with an increase in H3 acetylation at the *Bdnf* promoter observed in both the sperm of the sires and the mPFC of the offspring (Vassoler et al., 2012). Consistent with this, male offspring of cocaine-exposed fathers exhibited increased expression of *Bdnf*, which was found to suppress cocaine seeking behavior (Berglind et al., 2007).

Similarly, alcohol consumption was decreased in male offspring after paternal alcohol exposure (Finegersh and Homanics, 2014; Rompala et al., 2017). Persistent hypomethylation at the *Bdnf* promoter was detected in both the sperm of alcohol-exposed sires and the ventral tegmental area (VTA) of the offspring, accompanied by an increase in VTA *Bdnf* expression, which was implicated in alcohol preference and sensitivity (Moonat et al., 2013; Ting-A-Kee et al., 2013; Raivio et al., 2014).

These studies, while limited in number, suggest that heritable epigenetic modification induced by environmental exposure affects cellular and behavioral responses to addictive substances in the offspring, However, it is noteworthy that none of these epigenetic studies examined substance exposure prior to spermatogenesis or effects beyond the F1 generation. Therefore, the extent to which paternal substance exposure induces epigenetic changes that are stably maintained in the germline requires further inquiry.

#### 3.1.2 Gestational substance exposure

Most addictive substances and/or their metabolites can pass through the placenta (Rosen and Johnson, 1988; Schenker et al., 1993), interfering with brain development including the reward signaling system. Consequently, infants born to substance-dependent mothers are at risk for developmental and neurobehavioral deficits (Lesser-Katz, 1982). Furthermore neonates with gestational substance exposure can display withdrawal syndromes due to the abrupt cessation of substance exposure upon birth (Nichols, 1967; Vagnarelli et al., 2006; Patrick et al., 2020). Moreover, gestational substance exposure can lead to neuroinflammation and elevated permeability of the blood-brain barrier, increasing vulnerability to future toxin exposure (Kousik et al., 2012). These various outcomes could contribute to increased vulnerability to substance use later in life.

Two rodent models support the hypothesis that gestational substance exposure increases adult SUD vulnerability through epigenetic regulation, although causality has not been fully demonstrated. Δ-9-tetrahydrocannabinol (THC) exposure during pregnancy increased morphine-CPP in adult offspring, accompanied by a decrease of DRD2 receptor density in the NAc. Strikingly, this was also observed in aborted human fetal brain tissue from THC-consuming mothers. Further examination revealed an increase in the repressive H3K9me2 mark, a reduction in the permissive H3K4me3 mark, and a reduction in RNA polymerase binding at the *Drd2* gene. All three mechanisms may contribute in combination to downregulation of the DRD2 receptor (Dinieri et al., 2011; Sadeghzadeh et al., 2017). A change in the balance between permissive and repressive epigenetic marks was also associated with increased cocaine vulnerability in a mouse model of gestational methamphetamine (METH) exposure (Itzhak et al., 2014). Gestational METH exposure altered DNA methylation in the hippocampi of adult offspring, and increased cocaine-CPP and locomotor sensitization. Interestingly, these effects on DNA methylation opposed histone modifications at the same site. For example, DNA hypomethylation (permissive) was observed at the same sites modified by H3K27me3 (repressive), and DNA hypermethylation (repressive) was found at H3K4me3 (permissive) modified sites. The co-existence of these counteracting regulatory mechanisms highlights a complex balance of epigenetic modifications that underlie behavior abnormalities.

#### 3.1.3 Adolescent substance use

Adolescence is a second critical period when the brain undergoes rapid development and changes in plasticity, resulting in further maturation of higher-order executive and cognitive abilities (Aoki et al., 2017; Larsen and Luna, 2018). External perturbations during this period, including the use of addictive substances, increase vulnerability to psychiatric disorders, such as depression, anxiety, and SUDs (Reynolds et al., 2019; Volkow and Wargo, 2022). Adults with a history of adolescent substance use are more likely to acquire long-term, severe substance use problems (Breslau et al., 1993; Odgers et al., 2008; McCabe et al., 2022). These observations suggest that addictive substances produce distinct effects on brain development during adolescence, which may contribute to vulnerability to SUDs in adulthood.

Adolescent alcohol consumption increased alcohol-preference in adulthood in rodent models through epigenetic regulation. Kyzar et al. (2019) demonstrated that adolescent alcohol exposure increased miR-137 levels in the adult rat amygdala, which downregulated its target, lysine-specific demethylase 1 (LSD1). This resulted in enrichment of H3K9me2 (repressive) at the *Bdnf4* promoter, thereby decreasing *Bdnf4* transcription. Demonstrating its functional significance, intra-amygdala infusion of miR-137 antagomir rescued behavioral, transcriptional, and epigenetic changes induced by adolescent alcohol exposure, whereas intra-amygdala knockdown of LSD1 diminished the rescuing effects of miR-137 antagomir. In contrast, adolescent alcohol exposure in mice increased histone H4 acetylation (a permissive mark) at the *Bdnf* promoter and *Bdnf* transcription in the mPFC (Montesinos et al., 2016). These transcriptional and epigenetic changes were reversed by blocking the toll-like receptor 4 (TLR4) pathway, normalizing alcohol-preference in adults. Adolescent alcohol exposure also elevated drinking behavior in adult rats through altered enrichment of H3K27ac in the amygdala, since targeted change of this chromatin mark ameliorated excessive drinking in adulthood (Bohnsack et al., 2022). Taken together, these studies suggest that adolescent alcohol exposure induces changes in the epigenome, gene expression, and increased vulnerability to SUD in adulthood.

### 3.2 Stress

Severe acute or chronic stress induced by unpredictable or uncontrollable adverse events causes molecular, cellular, cognitive and behavioral abnormalities (Mineka and Kihlstrom, 1978; Taylor, 2010). Children who have experienced early-life adverse events such as bullying are more likely to use addictive substances in adulthood (Ttofi et al., 2016). More specifically, exposure to adverse childhood experiences is proportionally associated with increased odds of lifetime substance abuse, early initiation, and low cessation rates (Zarse et al., 2019). In adults, post-traumatic stress disorder is associated with increased chances of developing short-term or lifetime SUDs (Goldstein et al., 2016), while perceived work-related stress exhibits a positive correlation with alcohol and tobacco dependence (Peretti-Watel et al., 2009).

#### 3.2.1 Early-life stress (ELS)

Early-life stress has been modeled in rodents through maternal separation or low-quality maternal care (e.g., fostering, early weaning, limited bedding/nesting material) (Murthy and Gould, 2018). Consistent with the substantial body of evidence in clinical studies that ELS alters DNA methylation (Kinnally et al., 2011; Szyf and Bick, 2013; Catale et al., 2020), research with animal models also implicates DNA methylation in conferring vulnerability to adult SUDs after ELS. Maternal separation enhanced behavioral sensitivity to cocaine in adult rat offspring, along with hypermethylation of the *Pp1c* gene promoter in the NAc (Anier et al., 2014). These molecular and behavioral effects were mediated at least in part by increased expression of DNMT and were reversed by DNMT inhibition (Anier et al., 2010). In contrast, recipients of high-quality maternal care (Bilbo et al., 2007) exhibited lower morphine-CPP in adulthood compared to those receiving standard maternal care. The same group found decreased DNA methylation at the promoter of the anti-inflammatory cytokine IL-10 coding gene (*Il10*), which increased IL-10 expression in the NAc, and protected against morphine-induced neuroinflammation in glial cells (Schwarz et al., 2011). Thus, these findings implicate DNA methylation as a potential mediator between ELS and susceptibility to SUDs.

#### 3.2.2 Chronic stress in adulthood

Drug-naïve adult animals exposed to chronic stress subsequently display increased vulnerability to SUD-like behavior, accompanied by stress-induced epigenetic changes. Chronic stress significantly enhanced morphine-CPP 24 h after repeated daily foot shock in adult male rats. Striatal enrichment of the permissive mark H3K4me2 at the *Fosb* promoter was elevated in the shocked group prior to morphine exposure, which potentiated the expression of *Fosb* during the morphine-CPP sessions. These effects may be mediated by the glucocorticoid receptor (GR) signaling pathway, since co-administration of the GR antagonist mifepristone diminished or normalized behavioral, transcriptional, and epigenetic alterations produced by the chronic foot shock (Chen et al., 2019). This suggests that chronic stress exposure sensitizes responses to opioids through alterations in GR-mediated epigenetic regulation.

### 3.3 Physical activity

Physical exercise mitigates many psychological disorders and generally improves the quality of life (Fossati et al., 2021). Physical exercise in SUD treatment programs has been shown to reduce drug craving, ease symptoms of withdrawal, reduce depression and anxiety, and increase rates of abstinence (Wang et al., 2014; Dai et al., 2020). Preclinical studies reveal that physical activity is intrinsically rewarding, potentially serving as an alternative reinforcer to addictive substances (Cosgrove et al., 2002; Greenwood et al., 2011; Robertson et al., 2016; Robison et al., 2018).

In animal models, physical exercise regulates key genes involved in SUD-like behavior. Peterson et al. (2014) reported that physical activity dose-dependently reduced rats’ cocaine-seeking behavior and *Bdnf4* expression in the PFC, potentially through a reduction of histone acetylation. Since cocaine induces *Bdnf* expression (McCarthy et al., 2012), which in turn enhances cocaine-induced locomotor activity and cocaine SA (Schoenbaum et al., 2007), physical exercise may epigenetically counteract the overexpression of *Bdnf4* in the PFC and therefore mitigate the addictive potency of cocaine.

### 3.4 Conclusion

An individual’s interactions with external factors such as previous substance use, stress, and physical activity, can affect their vulnerability to future SUDs. These changes in SUD vulnerability have been proposed to involve the kinds of the long-term changes in regulation of the genome that occur in human and animals after prior experiences (Cadet, 2016; Ajonijebu et al., 2017; Hamilton and Nestler, 2019). And yet, as reviewed here, direct evidence to support this contention is surprisingly limited. This likely reflects the practical challenges in empirically demonstrating the mediating effects of epigenetic mechanisms on the relationship between prior environmental events and subsequent SUD-related behavior. To do so effectively, it is critical to design models that capture environmental factors and SUDs, ands to select appropriate transcriptional and epigenetic targets for validation. Given the likely role of prior environmental exposure in the development of SUDs, further elaboration of the manner in which this occurs would be invaluable. For example, most studies reviewed in this section could benefit from targeted epigenetic editing (further discussed in the next section) at genes of interest to further strengthen the case for a causal link between prior life events and later vulnerability to SUD-like behaviors. Nevertheless, even from our limited dataset, some intriguing findings have emerged. Most notably, and contrary to expectations, paternal preconception drug exposure epigenetically reduced male offspring’s drug taking in several studies (Vassoler et al., 2012; Finegersh and Homanics, 2014; Rompala et al., 2017).

## 4 Discussion

Our review has underscored both the sparsity and inconsistency of findings on the role of epigenetic regulation on SUD-related behavior and on precisely how it mediates environmentally predisposed SUD vulnerability. We conclude by offering several directions for future research that may aid in further elaborating the epigenetic landscape that lies between prior experience and development of SUDs.

### 4.1 Need for systematic and coordinated preclinical research on epigenetic mechanisms of SUD

As reviewed in section 2 “Epigenetic modifications mediating addictive effects of substances,” one prominent issue with preclinical research on epigenetic mechanisms underlying SUD is the inconsistency of findings regarding the functions of each epigenetic modification. This is not surprising given the molecular complexity of epigenetic regulation and the experimental designs that vary across studies. However, without a more detailed understanding of how the various forms of modification contribute to SUD vulnerability, our characterization of environmental influences and our search for new therapeutic tools to mitigate them will remain elusive. To these ends, it would be beneficial to adopt a more constrained set of parameters, and thereby increase comparability, across studies. For example, among the studies reviewed in section 2 “Epigenetic modifications mediating addictive effects of substances,” animals’ SUD-related behaviors were modeled by 4 measures in the CPP paradigm and 12 measures in the SA paradigm. While the choices of SUD-related measures in individual studies are no doubt grounded in sound rationales, findings would be more easily integrated if researchers were to prioritize the use of behaviors that show relatively robust differential responses to epigenetic manipulations, such as the acquisition phase in CPP and the drug-induced reinstatement phase in the SA paradigm. Furthermore, most of the current mechanistic studies focus on a single level of epigenetic modification. Therefore, little is known about how different mechanisms interact in producing a particular outcome.

### 4.2 Need for further preclinical studies on environment-induced epigenetic regulation in SUDs

Given the necessarily correlative nature of most human studies, animal studies offer the best opportunity to tease apart causal relationships between adversity, the epigenome, and later SUD-like behavior. That said, it is surprising how few studies have taken full advantage of animal models to establish causal relationships between environmentally induced epigenetic changes and their long-term effects on SUD vulnerability. First off, only a small number of animal models that capture environmentally induced epigenetic regulation have been employed in SUD studies. For example, no studies have evaluated effects of acute-stress induced epigenetic changes on subsequent SUD vulnerability in animal models. However, both clinical and preclinical studies have reported that acute stress alters the epigenome and may also regulate long-term SUD vulnerability (Vaisvaser et al., 2016; Carter et al., 2017; Rusconi and Battaglioli, 2018). In addition, early-life nutritional status mediates psychiatric outcomes later in life, including schizophrenia, major affective disorder and personality disorders (Brown et al., 2000; Xu et al., 2009; Hock et al., 2018). For example, prenatal folic acid (a methyl donor) supplementation shows promise in alleviating fetal alcohol spectrum disorder (Gupta et al., 2016). Although injections of the methyl donors MET and SAM altered animals’ addiction-like responses to cocaine (see section 2.1.2 “Behavioral effects”), dietary methyl donor supplementation (e.g., choline) that has more clinical relevance has not been tested. Moreover, a limited number of addictive substances have been examined in each paradigm. Stimulants, especially cocaine, have been the most studied class of substances, followed by alcohol. In contrast, findings regarding opioids and nicotine are relatively sparse. This is unfortunate, given the former being the most lethal and the latter the most prevalent of abused substances (Reynolds et al., 2021; United Nations Office on Drugs and Crime, 2023). Given the compelling evidence that environmental factors and life experiences induce substantial epigenetic modifications, which may in turn alter molecular, cellular, and behavioral processes, it is past time to expand the range of SUDs investigated in these epigenetic/behavioral paradigms.

### 4.3 Need for advanced functional and mechanistic studies

Many studies have documented the impact of environmental exposure on gene regulation and expression in reward signaling pathways in human SUDs or animal models (Szutorisz and Hurd, 2018; Walters and Kosten, 2019; Wanner et al., 2019; Smith et al., 2020; Swenson et al., 2020; Siomek-Gorecka et al., 2021). Nevertheless, their contribution to our understanding of underlying mechanisms has been limited. Many animal studies on the relations between epigenetic mechanisms and SUD vulnerability have focused on the epigenetic effects of substance exposure *per se*, rather than the effects of these epigenetic marks on future SUD vulnerability. Similarly, most studies that have associated prior environmental exposure with subsequent SUD by comparing their shared transcriptomic and epigenomic effects have not established a causal relationship. Evidence of correlations also does not help in elucidating whether substance-induced epigenetic changes are adaptive, or further, protective responses against adverse exposures or dysfunctional alterations that elevate SUD risks in the future (Rogers and Leslie, 2024). Therefore, the field would benefit from more studies explicitly designed to identify causal epigenetic relationships between environmental factors and SUD-related behavioral outcomes. For example, epigenetic changes induced by early-life events could be manipulated prior to SUD-like behavioral tests in animal models to confirm their functions in mediating SUD vulnerability. More detailed epigenetic regulatory mechanisms that mediate these processes could be investigated using cell-type specific gene knockdown or overexpression (Colby et al., 2003; Yamaguchi et al., 2018). Modifying DNA methylation and histone phosphorylation have emerged thus far as most consistently affecting relevant behaviors and could be prioritized in such investigations.

Newly developed techniques for targeted epigenome editing promise to accelerate the discovery of the details of epigenetic regulation underling SUD vulnerability. As with targeted editing of the genome, the epigenome can be precisely modified by introducing a fusion protein that contains an epigenetic catalyzing domain and a DNA-recognizing and -binding complex. The most commonly used platform is the clustered regularly interspaced short palindromic repeat (CRISPR)/Cas9 system. Fusing epigenetic modifiers (e.g., DNMTs, TETs, HATs, HMTs) to nuclease-deactivated Cas9 (dCas9) protein allows the epigenetic modifiers to be directed to the target sites by single-guide RNA (sgRNA) where they alter local epigenomic marks and regulate gene activity (Gjaltema and Rots, 2020). Compared to broad-based modification of epigenetic marks by means of viral vectors or perturbations of enzymatic activity, epigenome editing offers the prospect of more subtle interrogation of molecular targets and regulatory pathways implicated in correlational studies.

### 4.4 Comprehensive epigenetic profiling

Studies reviewed in section 3 “Environment and vulnerability to SUDs: role of epigenetics” primarily examined epigenetic marks and their gene targets that have been implicated in earlier addiction research, such as *Fosb*, *Bdnf*, and *Drd2* (Larson et al., 2010; Gorwood et al., 2012; Li and Wolf, 2015). While examining molecules that are known to be involved in SUDs could further validate earlier findings, this does not offer new insights into the role of the vast number of genes and gene networks that have not been investigated in SUD research. On the other hand, epigenetic sequencing techniques that have matured in recent years (e.g., CUT&RUN, CUT&Tag, Third-generation sequencing) allow for comprehensive assessments of genomic sites that are regulated by epigenetic marks of interest. In addition, the studies reviewed above investigated changes in epigenetic modifications and their targeted genes in “bulk” brain tissue (i.e., undifferentiated according to cell type), therefore preventing dissection of regulatory processes specific to each cell type. To this end, single-cell transcriptomic and epigenomic sequencing offers the opportunity to profile high-resolution, cell type-specific epigenetic changes (Mazan-Mamczarz et al., 2022). Comprehensive epigenetic profiling provides opportunities to discover new gene targets and gene networks that are regulated by environmentally induced epigenetic changes and may influence subsequent SUD vulnerability.

### 4.5 Employing behavioral predictors of SUD vulnerability

Similar to human populations, lab animals exhibit different levels of vulnerability to addictive substances due to differential genetic composition and environmental exposures (Solberg Woods and Palmer, 2019; Carrette et al., 2021). However, vulnerability in these studies is typically evaluated retrospectively by degree of drug taking, sometimes indexed across multiple measures (Navandar et al., 2021; Kaplan et al., 2022). This leaves open the possibility that epigenetic differences identified between vulnerable and resilient animals are a consequence of differential drug consumption and not a cause of it. Therefore, it would be beneficial to further explore prospective measures of SUD vulnerability that do not themselves involve drug exposure or that equate drug exposure across subjects (Swain et al., 2021). One exciting recent development is UCSD’s “RATTACA” project, which can provide researchers with genetically diverse Heterogenous Stock rats that have been selected for a behavioral trait, such as vulnerability to a given form of SUD, based on their global genotypic profile (Johnson et al., 2023).

### 4.6 Integration of preclinical and clinical studies

The rationale for conducting animal studies of SUD and other pathological disorders is to further our understanding of the human conditions such studies seek to model. But this depends on both the construct validity of a given behavioral paradigm and the extent to which the brain’s anatomical, physiological and molecular milieux are conserved across species, neither of which can be taken for granted (Everitt et al., 2018; Rydell-Törmänen and Johnson, 2019; Uliana et al., 2022). Opportunities to test the clinical relevance of findings from the animal lab are limited, which means that considerable resources may be invested in studying mechanisms that may be ultimately of little clinical significance. This fundamental problem can be mitigated by selecting epigenetic targets that are deemed more likely to apply across species on *a priori* grounds. Such filtering can be facilitated, at least to a degree, through statistical integration of the results of large-scale genomic and epigenomic studies that have been conducted in parallel across humans and other species. Thus, overlapping lists of genes differentially expressed in humans and other species exposed to opioids have been compared through Rank-rank Hypergeometric Overlap analysis (Liu et al., 2021; Browne et al., 2023), a threshold-free algorithm to calculate concordance between two complete gene expression profiles (Plaisier et al., 2010). More recently, a comprehensive platform, Mergeomics, has been developed that can utilize a full spectrum of multiomic datasets across species, as well as across tissue and cell types, epigenetic marks and genotypes, to reveal commonalities in gene networks and molecular pathways underlying pathophysiology in humans and other species (Shu et al., 2016; Ding et al., 2021).

### 4.7 Conclusion

Given the associations between environmental adversity and future SUD vulnerability, it is critical to understand the molecular mechanisms that mediate them. Epigenetic modifications are primary candidates since they are sensitive to environmental stimuli as well as genetic variation, can be long-lasting and, as reviewed here, can impact SUD-related behaviors in preclinical models. While current theories are still largely inferential, the adoption of novel and more systematic approaches in preclinical models will allow more precise and comprehensive characterization of the role of interacting epigenetic mechanisms in vulnerability to SUD, from regulation of individual genes to reshaping gene expression networks and brain circuitry. This, in turn, will yield dividends in the development of novel interventions to mitigate the devastating impact of addictive drugs on individuals and society.
